# Prevalence and risk factors for non-suicidal self-injury among patients with depression or bipolar disorder in China

**DOI:** 10.1186/s12888-021-03392-y

**Published:** 2021-08-04

**Authors:** Lu Wang, Jun Liu, Yuan Yang, Haiou Zou

**Affiliations:** 1 School of Nursing, Anhui Medical University, No.69 Mei Shan Road, Shu Shan District, Hefei, 230031 Anhui Province China; 2 Department of Nursing, Beijing An Ding Hospital, 5 Ankang Lane, Xicheng District, Beijing, 100088 China; 3 School of Nursing, Peking Union Medical College, Badachu Road, Shijingshan District, Beijing, 100144 China

**Keywords:** Non-suicidal self-injury, Prevalence, Risk factors, Depression, Bipolar disorder, China

## Abstract

**Backgrounds:**

Non-suicidal self-injury is a serious health problem among patients with depression or bipolar disorder. However, few studies within the Chinese context have investigated the prevalence of NSSI and its risk factors in above populations. The purpose of this study was to investigate the prevalence of non-suicidal self-injury and its risk factors in patients with depression or bipolar disorder in China.

**Methods:**

The final sample comprised of 394 inpatients(*M*_*age*_ = 29.71; *SD*_*age*_ = 11.95) with depression or bipolar disorder from two psychiatric hospitals in Beijing, China. A General Demographic Data Form, the Non-suicidal Self-injury Questionnaire(NSSI-Q), Impulsivity Item and the Adverse Childhood Experiences-International Questionnaire(ACE-IQ) were completed by all patients.

**Results:**

Of the 394 patients examined, 245(62.2%) of this sample reported NSSI in past year. Of the 245 patients with NSSI, 135(55.1%) were diagnosed with depression and 110(44.9%) were diagnosed with bipolar disorder. The most common methods of NSSI for female was “pinching”(23.1%) and “scratching”(22.8%), while for male it was “hiting hard objects”(12.7%). By multivariate regression analysis, young age, unemployment, a higher monthly family income, single, impulsivity, long duration of illness and ACEs were risk factors for NSSI in patients with depression and bipolar disorder(*P*<0.05).

**Conclusions:**

Our study points to the fact that there was an unfortunate message about the prevalence of NSSI among patients with depression or bipolar disorder in China. It is necessary not only to raise the awareness of NSSI in families and society, but also to formulate targeted assessment and intervention. Moreover, future research should not only focus on individuals being hospitalized, but should be representative of individuals treated at home or in the community because there are no national statistics on NSSI among such patients in China.

## Background

Non-suicidal self-injury(NSSI) is generally defined as “the direct, deliberate destruction of one’s own body tissue without the intention of suicidal intent” [1], including cutting or scratching the skin, burning/branding with cigarettes/lighters, scalding, striking oneself or other hard objects, banging limbs/head and hair pulling, et al. [2]. Based on previous studies, DSM-5 provided a more accurate definition of NSSI, that is, in the last year, an individual has been engaged in a behavior that intentionally causes bleeding, bruising, or pain on the body surface for 5 or more days, but only causes slight or moderate physical injury [3]. As one of the public health problems recognized globally, NSSI has provoked concern among health professionals, researchers, social workers and welfare workers, teachers, other professionals and affected families [4].

Estimated prevalence of NSSI vary widely as due to a number of factors including the time since last episode of NSSI, the number of NSSI episodes to be recognized, research tools, as well as different study areas and populations [5]. Benjet et al. found that the lifetime prevalence of NSSI was 18.56%, and the annual prevalence was 3.19% in a sample of 1071 Mexican residents of young adults [6]. In Canada, estimated prevalence range from as low as 7% [7] of student samples to as high as 77% [8] of clinical samples. As a coping strategy for maladaptive individuals [9], NSSI are more prone to be seen in clinical populations, especially in patients with depression or bipolar disorder who have poor abilities of emotional regulation and coping [10]. A recent study observed that about 37 and 52% of patients with depression and bipolar disorder had engaged in NSSI at least once, respectively [11]. Fang et al. found that 38.6% of Chinese patients with depression had committed NSSI in the past year [12]. Although there have been a few relevant studies in China, the evidence on the prevalence of NSSI is still sparse and heterogeneous due to the differences in sample sources and definitions of NSSI [13, 14]. It is necessary to carefully design studies to help better understand the epidemiology of NSSI in Chinese patients with depression or bipolar disorder.

NSSI leads to a variety of serious consequences, including physical injury [15], negative emotional experiences and a decline in learning ability, work efficiency [16], which prompted researchers to explore the risk factors relate to NSSI. A study proposed that young individuals were more prone to conduct NSSI [17]. However, the evidence on whether NSSI changes with age is insufficient. Moreover, there is contradictory evidence about whether there have a sex difference in NSSI with some studies reporting a higher prevalence among female [18] and others finding no sex difference [19]. Also, NSSI was uniquely associated with marital status, employment status, socio-economic status. Nevertheless, findings should be cited with caution [20]. Tschan et al. presented that the educational level of parents was relate to NSSI [21]. Besides, there may also be a link between impulsivity and NSSI [11], which may be more pronounced in individuals with depression or bipolar disorder than in the general population [22]. A new study revealed that cannabinoid use was connected with an increased prevalence of NSSI by elevating Δ^9^- THC/CBD balance [23], which makes us to think about the relationship between substance abuse experiences such as alcohol, cannabinoid and NSSI. To date, the existing literature is inconsistent with regard to whether the incidence of NSSI is related to adverse childhood experiences(ACEs). In earlier times, Glassman have failed to find a significant association between NSSI and ACEs [24]. However, new studies provided new clues. In a meta-analysis that included cross-sectional, longitudinal, and retrospective studies published in the same year, Serafini et al. observed that the increased vulnerability to NSSI seems to be related to ACEs [25].

Whereas studies from other countries have investigated the prevalence of NSSI and risk factors of NSSI [20, 25, 26], including the exploration of patients with depression or bipolar disorder [5, 11]. So far, few studies within the Chinese context have investigated the prevalence of NSSI and the risk factors for NSSI among patients with depression or bipolar disorder. Almost all of the few existing studies on NSSI in China are from the general population or relied on student samples where the characteristics are far from representative of patients with depression or bipolar disorder. The purpose of this study aimd to: 1) examine the prevalence of NSSI in patients with depression or bipolar disorder in China, and 2) determine the risk factors for NSSI.

## Methods

### Participants

The participants for this cross-sectional study consisted of patients who were hospitalized at one of the two Chinese biggest psychiatric hospitals in Beijing(Beijing Anding Hospital and Beijing HuiLongGuan Hospital), China, between September 2019 and May 2020. Inclusion criteria were: 1) The patients who were aged 18 to 60 years old were interviewed by two or more psychiatrists and met the diagnostic of depression or bipolar disorder according to the International Statistical Classification of Diseases and Related Health Problems, Tenth Revision (ICD-10); 2) The patients were clinically stable(an increase in drug dosage not more than 50% in the 3 months before this investigation [27]); 3) The patients were able to read and write in simplified Chinese; 4) The patients were willing to join our study after providing informed consent. Exclusion criteria were the presence of any condition that may affect the ability to complete the questionnaire and the accuracy of the results, including accompanied by other mental illnesses, delirium, dementia, deafness, mental retardation or denial of informed consent.

### Procedures

From September 2019 to May 2020, 412 adult patients were eligible for our study. Of these patients, 16 cases were excluded from the analysis because of the unwillingness to continue to participate in study. The final sample consisted of 394 patients, with a a response rate of 95.63%. Before entering our study, we explained the purpose, content and procedure of this study to the patients, and promised that the study results would only be used for academic research. Patients voluntarily decide whether to participate in the study or not. If they agree to participate, they will sign a written informed consent. Moreover, patients have the right to decide whether to continue during the study. The questionnaire information was collected face-to-face, one-on-one, by trained researchers in a quiet room in the ward after being given a verbal and written explanation of the study and having acquired informed consent. And the questionnaire was completed by themselves. If the respondents have questions about the questionnaire, the researchers can help them understand by informing them of the original intention of the question in a non-judgmental way. Additionally, the quality control procedures in the research process are also very important. Measures were taken for quality assurance, such as intensive researcher training, detailed field explanations, continuous feedback and independent supervision of supervisors and field researchers. The questionnaire was collected and checked whether there were any missing items by the researcher on the spot after completion. The study was performed according to the Declaration of Helsinki and approved by the Research Ethics Board of Peking Union Medical College (2019–18-K7).

### Instruments

#### Demographic details

A General Demographic Data Form by self-designed was employed to collect basic information of each patient, including sex, age, marital status, employment status, monthly family income, residence, educational level of patients and their parent, left-behind experience, substance abuse experience, family type, family structure, and the clinical features of patients(details see Table 1).

**Table 1:** Demographic characteristics, implusivity and ACEs between Groups (*n* = 394)

	(n, %)			Comparisons	
	D-NSSI Group	BD-NSSI Group	Non-NSSI Group	χ^2^	*P*-value
**Participants**	135(34.3)	110(27.9)	149(37.8)		
**Age (years)**				67.348	< 0.001
18 ~ 30	110(81.5)	80(72.7)	55(36.9)		
31 ~ 45	17(12.6)	21(19.1)	61(40.9)		
46 ~ 60	8(5.9)	9(8.2)	33(22.2)		
**Sex**				0.559	0.756
Male	38(28.1)	33(30.0)	48(32.2)		
Female	97(71.9)	77(70.0)	101(67.8)		
**Education of patients**				1.586	0.811
Junior school graduate and below	18(13.3)	18(16.4)	17(11.4)		
High school graduate	29(21.5)	21(19.1)	34(22.8)		
University and above	88(65.2)	71(64.5)	98(65.8)		
**Employments status**				25.439	< 0.001
Employed	40(29.6)	55(50.0)	88(59.1)		
Unemployed	95(70.4)	55(50.0)	61(40.9)		
**Marital status**				60.204	< 0.001
Single	107(79.3)	79(71.8)	57(38.2)		
Married	22(16.3)	23(20.9)	80(53.7)		
Divorced or widowed	6(4.4)	8(7.3)	12(8.1)		
**Monthly family income (US dollar)**				11.212	0.024
400 ~ 700	31(23.0)	33(30.0)	55(36.9)		
700 ~ 1000	47(34.8)	24(21.8)	43(28.8)		
> 1000	57(42.2)	53(48.2)	51(34.3)		
**Residence**				1.248	0.536
Urban	86(63.7)	77(70.0)	96(64.4)		
Rural	49(36.3)	33(30.0)	53(35.6)		
**Left-behind experience**				5.113	0.078
Yes	39(28.9)	32(29.1)	28(18.8)		
No	96(71.1)	78(70.9)	121(81.2)		
**Substance abuse experience**				8.638	0.013
Yes	27(20.0)	30(27.3)	19(12.8)		
No	108(80.0)	80(72.7)	130(87.2)		
**Family type**				0.754	0.945
Nuclear family	72(53.3)	60(54.5)	86(57.7)		
Joint family	25(18.5)	18(16.4)	24(16.1)		
Single family	38(28.2)	32(29.1)	39(26.2)		
**Family structure**				1.205	0.547
Single child family	68(50.4)	60(54.5)	71(47.7)		
Multiple children family	67(49.6)	50(45.5)	78(52.3)		
**Education of father**				6.071	0.194
Junior school graduate and below	64(47.4)	44(40.0)	73(49.0)		
High school graduate	21(15.6)	20(18.2)	33(2.2)		
University and above	50(37.0)	46(41.8)	43(28.8)		
**Education of mother**				5.688	0.224
Junior school graduate and below	61(45.2)	45(40.9)	82(55.0)		
High school graduate	30(22.2)	28(25.5)	28(18.8)		
University and above	44(32.6)	37(33.6)	39(26.2)		
**Duration of illness (years)**				63.705	<0.001
< 3	108(80.0)	35(31.8)	86(57.7)		
3 ~ 5	16(11.9)	27(24.5)	19(12.7)		
6 ~ 10	5(3.7)	29(26.4)	22(14.8)		
> 10	6(4.4)	19(12.3)	22(14.8)		
**Times of recurrence**				26.002	0.001
0	45(33.4)	15(13.6)	42(28.2)		
1 ~ 3	64(47.4)	48(43.7)	62(41.6)		
4 ~ 5	13(9.6)	20(18.2)	26(17.5)		
> 5	13(9.6)	27(24.5)	19(12.7)		
**Impulsivity**				12.206	0.002
Yes	41(30.4)	43(39.1)	29(19.5)		
No	94(69.6)	67(60.9)	120(80.5)		
**Adverse childhood experiences**				26.313	<0.001
Yes	134(99.3)	107(97.3)	127(85.2)		
No	1(0.7)	3(2.7)	22(14.8)		

*D-NSSI Group* Depression-NSSI Group

*BD-NSSI Group* Bipolar Disorder-NSSI Group

#### Non-suicidal self-injury questionnaire(NSSI-q)

The Non-suicidal Self-injury Questionnaire was compiled by Wan et al. [28] in the investigation of nationwide large sample of NSSI. In 2018, Wan et al. [29] further developed the tool. There are 12 items in the questionnaire, which are divided into two dimensions: Items 1 to 7 involve NSSI without obvious tissue injury, which refers to that the NSSI carried out by individuals does not cause obvious and serious tissue damage; items 8 to 12 involve NSSI with obvious tissue injury, which refers to NSSI by individuals may result in bleeding, scratches and other tissue damage. Participants were asked to answer questions, “In the past year, have you ever engaged in the following behaviors to deliberately injure yourself but without suicidal intent?” The questionnaire investigated the occurrence of 12 categories of NSSI of the respondents in the past year, including pinching, scratching, hitting hard objects with head/fist, hitting themself with fists or hard objects, biting, pulling his/her hair, stabbing, cutting, scalding, etc. It is a 5-point Likert scale ranging from “never”(score 0) to “always”(score 4), with a total score of 0 ~ 48. A dichotomous variable of NSSI status was computed based on the 12 items. It was coded 0 when participants choosed “never” as the answer of all items, and was coded 1 when participants reported having engaged in one or more NSSI acts. NSSI-Q showed satisfactory reliability and validity as a self-report measure for NSSI [29]. In this study, the Cronbach’sαof the questionnaire was 0.82.

#### Impulsivity

Impulsivity was measured by the question: “Most of time in your life, no matter what the situation was, no matter who you were with, have you often done things impulsively?” Respondents who answered affirmatively were defined in this study as impulsive. For the assessment of impulsivity, it is derived from the borderline personality disorder assessment module using the *NIAAA Alcohol Use Disorder and Associated Disabilities Interview Schedule-IV* [30]*.* We cannot use test-retest reliability of individual items. However, the Cronbach’s alpha(0.77) for the borderline personality disorder assessment module was computed, indicating very good internal consistency of the section. After removing the impulsivity item, the value does not change, indicating that the item has high reliability [31]. Good convergent and divergent validity were previously embodied in this item [31].

#### Adverse childhood experiences-international questionnaire(ace-IQ)

ACEs were assessed using the Adverse Childhood Experiences International Questionnaire(ACE-IQ) developed by the World Health Organization [32], which covers 3 domains of childhood adversities, including childhood maltreatment, family/household dysfunction and violence outside the home. Collectively, these 3 domains on behalf of 13 categories of ACEs, which covers family dysfunction; physical, sexual and emotional abuse and neglect; peer violence; witnessing community violence, and exposure to collective violence. Each respondent was asked if they had experienced various adverse events during their childhood(prior to the age of 18). In this present study, the frequency scoring method was used to generate a conservative estimate of ACE exposure that closer to international standards. ACE scores were recorded as total counts for the number of questions to which respondents answered that they had experienced an ACE. The existence of any of the 13 categories of ACEs is considered to be the existence of ACEs. The ACE-IQ has good reliability and validity [33–36]. The Cronbach’sαof the questionnaire was 0.72 in this current study.

### Statistical analysis

Descriptive statistics for all the variables were first conducted using percentages for the categorical variables. Mean, standard deviation, and range were computed for continuous variables. The prevalence of NSSI was examined and presented in terms of frequency and the proportion of those encountering it(%). Social-demographic characteristics, impulsivity and ACEs of participants between NSSI group(Depression-NSSI group/Bipolar Disorder-NSSI group) and Non-NSSI group were compared by using Chi-squared tests. After running Chi-squared tests for each putative risk factor between groups (Table 1), we included in the multivariable logistic regression models those factors that showed a statistically significant difference(*P* < 0.05). In addition, based on clinical experiences and literature review, it was found that the sex was closely related to the occurrence of NSSI [37]. Therefore, sex was included in logistic regression for further analysis. All the variables were entered in the model using the enter method. All statistical differences were considered significant when the *P*<0.05 for both directions. All statistical analyses were performed in SPSS for Windows, Version 23.0.

## Results

The sample composed of 394 adult patients(Male = 119, Female = 275), with a mean age of 29.71 years(*SD* = 11.95). Male and female patients did not differ in age(29.81 ± 12.17 vs. 29.49 ± 11.46; *t* = − 0.25; *P* = 0.80). The response rate was 95.63%. Sixteen patients who were eligible for inclusion in this study declined to continue in the study. No patients returned incomplete or not analyzable questionnaires.

### The demographics, clinical features, impulsivity and ACEs

The average age of 394 respondents (119 male, 275 female) were 29.71 years, and they were from north China(65.5%), northeast China(7.9%), etc., most of them were female(69.8%). The majority of the respondents(65.2%) had finished university or above and 53.6% of the respondents were unemployed. In addition, the monthly family income of 161 participants exceeded 1000 dollars. More than half of the respondents(61.7%) were single and from urban areas(65.7%). 99(25.1%) and 76(19.3%) respondents had left-behind experience and substance abuse experience respectively. The educational level of the parents of the respondents was mostly university or above, especially father(35.3%). Two hundred eighteen participants(55.33%) were from the nuclear family. The number of participants from single child family(50.5%) and those from multiple children family(49.5%) accounted for about half each. Clinical diagnoses of the patients were depression(52.8%) and bipolar disorder(47.2%), of whom the majority were within 3 years of illness(58.1%) and within 1–3 times of recurrence(44.2%). 28.7% of participants reported impulsivity, and the vast majority of participants(93.4%) reported ACEs.

### The prevalence of NSSI and comparisons of demographics, clinical features, impulsivity and ACEs between groups

**Fig. 1:**
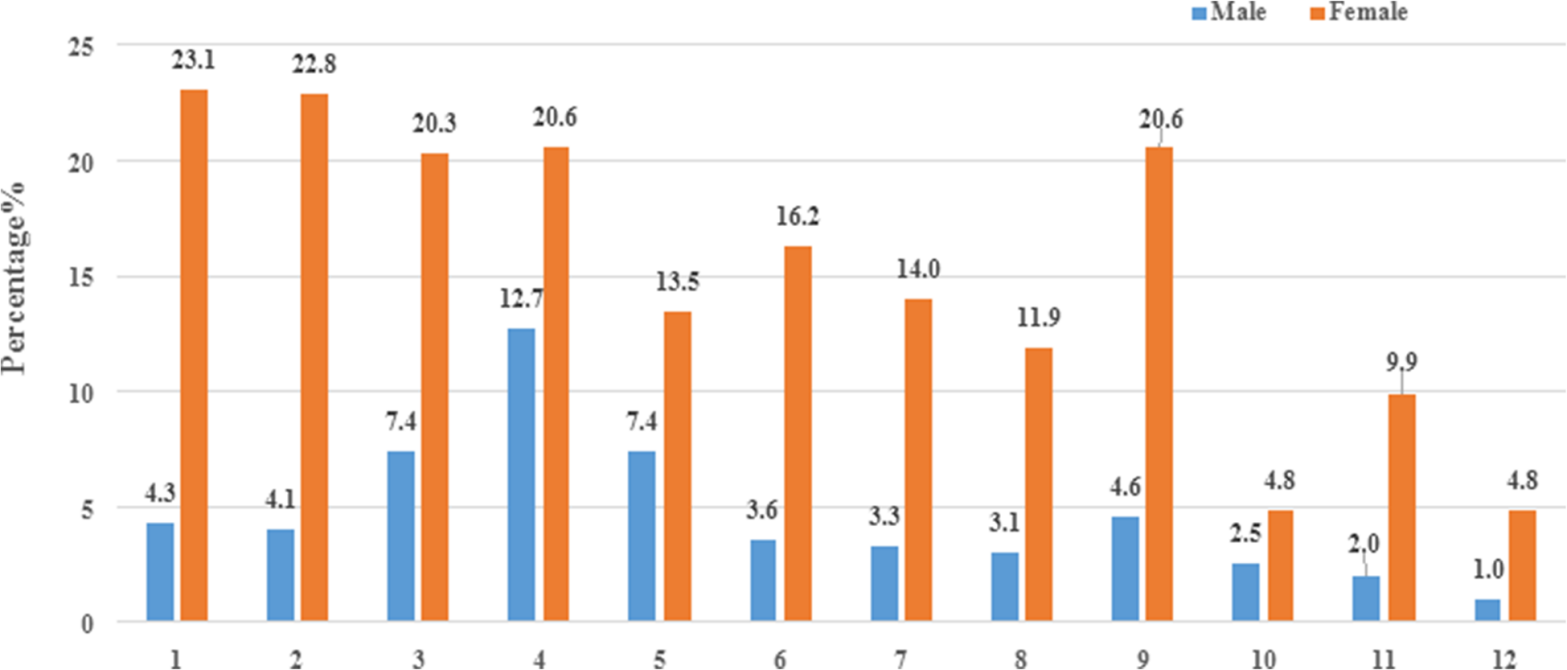
Frequency of 12 kinds of NSSI among male and female participants

The final sample comprised 245 adult patients accompanied with NSSI(62.2%) and 149 adult patients unaccompanied with NSSI(37.8%). Among 245 participants with NSSI, 135(55.1%) were diagnosed with depression and 110(44.9%) were diagnosed with bipolar disorder. Moreover, 57(14.4%) had engaged in one type of NSSI and 188(47.6%) had attempted more than one type of NSSI, respectively. “Deliberately pinching oneself”(23.1%) was the most common way of NSSI for female, while “Deliberately hiting hard objects such as walls, tables, windows and floors with fists”(12.7%) was the most common way of NSSI for male, as detailed in Fig.1.

We also compared the demographics and clinical features of patients in the Depression-NSSI group, Bipolar Disorder-NSSI group and the Non-NSSI group to explore the relationships. Chi-square test showed that age, employments status, marital status, monthly family income, substance abuse experience, duration of illness, times of recurrence, impulsivity and ACEs were statistically different between the three groups(all *P* < 0.05), as presented in Table 1.

### Risk factors associated with NSSI of participants

As revealed in Table 1, the variables that showed significance in the above comparison of 394 participants were further analyzed by multivariate regression analysis. As mentioned above, we also included sex in multivariate logistic regression based on the evidence from clinical and academic experience. By comparing patients between D-NSSI group and Non-NSSI group, we found increased risks of NSSI in depressed patients related to have a young age(*OR* = 0.300 and *OR* = 0.278; using 18 ~ 30 years old group as the reference category), unemployment(*OR* = 0.466; using unemployment group as the reference category), have a higher monthly family income(*OR* = 0.455; using > 1000 dollars group as the reference category) and have a history of ACEs(*OR* = 0.049; using ACEs group as the reference category), as presented in Table 2.

**Table 2:** Final multivariable model, comparing patients with D-NSSI Group and Non-NSSI Group

Variables	Odds Ratio	Standard Error	95% Confidence Interval	*P-*value
**Age (years)**				
18 ~ 30 ^a^	–	–	–	–
31 ~ 45	0.300	0.588	0.095–0.950	0.041
46 ~ 60	0.278	0.439	0.117–0.656	0.004
**Employments status**				
Unemployed ^a^	–	–	–	–
Employed	0.466	0.302	0.258–0.842	0.011
**Monthly family income (US dollar)**				
> 1000 ^a^	–	–	–	–
700 ~ 1000	0.935	0.349	0.472–1.852	0.848
400 ~ 700	0.455	0.351	0.229–0.906	0.025
**Adeverse childhood experiences**				
Yes ^a^	–	–	–	–
No	0.049	1.074	0.006–0.404	0.005

*D-NSSI Group* Depression-NSSI Group

*BD-NSSI Group* Bipolar Disorder-NSSI Group

^a^ Reference group

By comparing patients between BD-NSSI group and Non-NSSI group, 5 variables - age, marital status, impulsivity, ACEs and duration of illness - emerged as significant correlates of NSSI in patients with bipolar disorder. More specifically, in patients with bipolar disorder, the incidence of NSSI was significantly lower in 31 ~ 45 and 46 ~ 60 years old group(*OR* = 0.254 and *OR* = 0.326; using 18 ~ 30 years old group as the reference category). Next, low risks for NSSI remained associated with married status(*OR* = 0.318; using single group as the reference category), no impulsivity (*OR* = 0.415; using impulsivity group as the reference category) and no ACEs group(*OR* = 0.272; using ACEs group as the reference category). Finally, duration of illness for more than 10 years were more likely to have NSSI than those less than 3 years(*OR* = 0.255; using > 10 group as the reference category), as presented in Table 3.

**Table 3:** Final multivariable model, comparing patients with BD-NSSI Group and Non-NSSI Group

Variables	Odds Ratio	Standard Error	95% Confidence Interval	*P-*value
**Age (years)**				
18 ~ 30 ^a^	–	–	–	–
31 ~ 45	0.254	0.575	0.802–0.784	0.017
46 ~ 60	0.326	0.440	0.138–0.772	0.011
**Marital status**				
Single ^a^	–	–	–	–
Married	0.318	0.459	0.130–0.783	0.013
Divorced or widowed	0.710	0.613	0.213–2.361	0.576
**Impulsivity**				
Yes ^a^	–	–	–	–
No	0.415	0.339	0.214–0.808	0.010
**Adeverse childhood experiences**				
Yes ^a^	–	–	–	–
No	0.272	0.690	0.070–0.983	0.048
**Duration of illness (years)**				
< 3	0.255	0.516	0.093–0.702	0.008
3 ~ 5	0.932	0.529	0.330–2.631	0.894
6 ~ 10	1.173	0.504	0.437–3.154	0.751
> 10 ^a^	–	–	–	–

*D-NSSI Group* Depression-NSSI Group

*BD-NSSI Group* Bipolar Disorder-NSSI Group

^a^ Reference group

## Discussion

In terms of NSSI, there have been a few clinical studies involving patients with depression or bipolar disorder in Western countries [5, 38], however no such study was conducted in Chinese populations. This was the first study to investigated the prevalence of NSSI and risk factors for NSSI in patients with depression or bipolar disorder in China. Moreover, our findings indicated that a large number of patients with depression or bipolar disorder had engaged NSSI in this sample, and certain demographics, impulsivity and ACEs was associated with NSSI among patients with depression or bipolar disorder in China.

In the present study, our results of prevalence of NSSI was 62.2%, which was slightly lower than the prevalence of NSSI(77%) in a Canadian study among clinical populations [8]. Equally, Weintraub et al. [11] found that about 37% of patients with depression and 52% of patients with bipolar disorder had at least one NSSI, which was also higher than the results of this study(34.3 and 27.9%, respectively). The discrepancies in estimates of the prevalence of NSSI may be relate to different sample sources and numbers, various assessment tools, wording of instructions, time frame for raising questions and data collection procedures. As mentioned above, the incidence of NSSI is higher in patients with depression than in patients with bipolar disorder(34.3% vs 27.9%). Depression is a group of prominent and persistent low mood as the main features of the clinical syndrome [39]. Affected by the low mood, depressed patients often feel extremely sad, self abasement, decadent and pessimistic, and then the germ of an idea of NSSI took root in patient’s mind. In regard to the method of NSSI, patients of different sex tried NSSI in different ways. Barrocas [40] found ‘hitting against hard objects’ to be the most common way that male injury themselves, while Brunner [41] found ‘cutting’ to be the most common way that female injury themselves. These findings of ways of NSSI are generally consistent with this present study. Explainations for sex differences are yet to be examined and may be due to personality differences and cultural differences [42].

It has been suggested that demographic characteristics and clinical features(age, sex, marital status and diagnosis, etc) should be considered when interpreting results of any NSSI research [4]. Contrary to expectation, there was no statistical significance between patient’s sex, education, residence and the incidence of NSSI in patients with depression and bipolar disorder(*P*>0.05), which are contrary to Tang’s study [37]. On the one hand, this study included patients with depression and bipolar disorder. Compared with the general populations, their ability of emotion regulation and coping with life events is limited [43]. Whether male or female patients, the impact of negative life events and the failure of emotion regulation will stimulate the patients’ desire to conduct adopting stress reduction behaviors (e.g., NSSI), so as to escape from feelings and thoughts associated with stressful life events [44]. This may be the reason why there was no difference in the occurrence of NSSI between male and female patients in this study. On the other hand, this present study recruited patients from two psychiatric hospitals in Beijing, most of whom came from Beijing or the affluent urban families from other cities. Most included patients had better educational opportunities and higher level of education. The patients included in this study only reflect the situation of a small part of China, so the generalization of this finding is limited. Caution should be kept in quoting this findings.

To our surprise, statistical significance between substance abuse experience and the incidence of NSSI in patients with depression and bipolar disorder was not found(*P*>0.05), which was partially inconsistent with Escelsior’s findings [23]. This divergence is related to the fact that the substance abuse included in this study is not confined to cannabis, but also extended to various types of substance abuse. The number of patients with each type of substance abuse is limited, and the types of substance abuse are not concentrated, so we can not analyze all kinds of substance abuse experience separately, which may affect the results of this study. Notably, cannabis, inhalants, etc. for personal use are not allowed in Chinese laws [45], so the unwillingness of reporting such experiences hinders our further exploration. Different from other studies [46, 47], there is no significant difference in the left-behind experience between three groups, which may be related to the fact that most of the samples are from Beijing rather than remote and poor areas As the capital of China, Beijing has the ability to solve the employment problem without local people going out to look for employment opportunities [48], which was also reflected by the high educational level of patients and high monthly family income in this study.

By multivariate regression analysis, we proved that young, unemployment, single, a higher monthly family income, long duration of illness, impulsivity and ACEs were risk factors leading to NSSI of patients with depression and bipolar disorders. First of all, this study showed that younger participants(18 ~ 30 years old) had a higher risk of NSSI, which is consistent with Preyde’s findings that NSSI is more prevalent in young patients with psychiatric disorders due to their difficulty in emotional regulation, adaptive ability and interpersonal relationships [8, 49]. Next, the unemployed patients with depression were more likely to conduct NSSI. Compared with the employed patients, the unemployed patients can not have a relatively fixed incomes and stable personal relationships for a long time, which implied that it is inadequate for patients to get financial support and emotional support [50], increasing the risk of NSSI [51]. Moreover, we found increased risks of NSSI in depressed patients related to have a higher monthly family income. A higher monthly family income reflects that the family members who have limited spare time invested so much time and effort in work, which resulted in the limited time to get along with the patients, and then neglected the patients [52]. Due to the untimely response to the emotional needs of patients, the corresponding emotional support is insufficient, and patients are more likely to seek psychological comfort through NSSI. These findings highlight the importance of positive parenting style to NSSI onset, with implications for prevention of NSSI onset among depressed patients [53].

In this present study, marital status is one of the important factors affecting NSSI of patients with bipolar disorder, that is, single patients are more likely to engaged in NSSI, which has been repeatedly emphasized in previous studies [54–56]. It is widely known that family support obtained by stable marriage status was most salient in onset, maintenance and cessation of NSSI [55]. Stable marriage status provides social, economic and emotional support to patients and reduces their sense of isolation by providing them with opportunities to interact with society, and spouses of married patients play an important role in monitoring their partners’ health-related behaviors for a long time, encouraging them to develop healthy lifestyle [54]. Besides, long duration of illness(more than 10 years) was the risk factor for NSSI in patients with bipolar disorder. It is a long-term process to develop from unipolar depression to bipolar disorder [57]. During this period, the patient experienced repeated fluctuations of the disease. Affected by the symptoms or discontented treatment outcome, the patient felt hopeless and desperate, and then increased the possibility of NSSI. In addition, we proposed that impulsivity remained a significant influence on NSSI in patients with bipolar disorder, and this finding was supported by Lin et al. [58]. Individuals with strong impulsivity tend to act impulsively in the face of negative emotions, because the short-term gain of emotional regulation is the most important goal at present [59]. Since NSSI has been proved to be an effective method for individuals to regulate negtive emotions, individuals with strong impulsivity are more willing to participate in NSSI to obtain the direct benefits of NSSI(i.e. emotional regulation) [60]. At the same time, it is suggested that NSSI is a rapid, effective, and easily implemented method of regulating one’s negative emotion [58]. Consequently, impulsivity may be strongly related to NSSI.

In recent years, several studies found that individuals reporting ACEs were tied up with NSSI [61, 62], which were consistent with this study. What’s more, ACEs can be strongly associated to NSSI among patients with depression and bipolar disorder [5]. Although heritability is often emphasized, NSSI is associated with environmental factors [63]. As a series of negative life events, ACEs will affect the individual’s psychological development and emotional regulation [64]. And the patients with depression or bipolar disorder already have weak ability of emotional regulation [65]. Therefore, patients with ACEs can relieve their negative emotions by adopting NSSI. This also explains why ACEs has a subtle effect on the occurrence of NSSI in patients with depression and bipolar disorder. Serafini’s study also highlighted the specific role of sexual abuse in the development of NSSI [25], so exploring specific associations between various types of ACEs and NSSI would be the next step. So far, some existing studies have investigated the mediators or moderators in relationship between ACEs and NSSI [66]. It is necessary to explore the specific connections between ACEs and NSSI, especially in the Chinese backdround.

### Implications

This study investigated the prevalence of NSSI and its risk factors among patients with depression and bipolar disorder in China. Primarily, we have obtained the not encouraging prevalence to arouse the attention of domestic medical staff, relevant scholars and the public. That is to say, the coping strategies for NSSI should not be confined to hospitals but extended to the communities and families. Then, by comparing the demographic data, impulsivity and ACEs between groups, the relevant influencing factors were found, which can provide support for the exploration of prevention, treatment and etiology of this group. Eventually, the risk factors for NSSI was identified by multivariate regression analysis. Except for the assessment of general demographic data, assessment procedures of mental health may need to include an assessment of impulsivity and ACEs to more fully evaluate NSSI. A combination of the risk factors mentioned above and diagnosis of depression or bipolar disorder among patients who conduct NSSI can also alert medical staff to develop targeted interventions aimed at helping these patients and their families with emotion regulation skills so as to cope with past negative experiences and consequently improve their mental health and well-being. From an academic point of view, this study further confirmed the correlations between NSSI and some factors in patients with depression and bipolar disorder, further supported the conclusions of some scholars. This study also offered new thought for scholars of various contries to guide them to carry out more scientific and targeted research. Notably, future studies should focus on the origin of NSSI as opposed to its characteristics, in order for professionals to be able to prevent the issue.

### Limitations

Several limitations of the current study need to be acknowledged. Initially, the participants included were patients in two hospitals in Beijing. As the capital of China, Beijing will attract patients from all over the country, the samples are mainly Beijing native. Considering that China is a vast country with diversified social economy, the source of sample would limits the generalizability of these findings to other cities and rural areas of China. Second, we employed retrospective self-report questionnaires, and there is no independent confirmation, which might mean our data were not sufficiently objective. Participants may forget, suppress or even identify with their experiences as they grow older and more experienced. Therefore, these data may be affected by bias to some extent. Third, an exploration of testing the directionality of the relationships is not allowed in the present study because of the design of cross-sectional. There may be a great deal of analyses and the possibility that some of the correlations were obtained by chance. Taking into account these limitations, additional studies are needed to investigate the NSSI and tease apart the association between NSSI and some factors in order to determine how generalizable the results of this study would be to other psychiatric hospitals from diffirent regions in China. Such studies should employ a large sample, multicenter, longitudinal design, and adopt tools other than self-report questionnaire(e.g., expert opinions or other objective evidence). And we should remain cautious in drawing causal inferences regarding the relationship between NSSI and some factors.

## Conclusions

Despite these limitations, the current study examined the prevalence of NSSI and its risk factors in patients with depression and bipolar disorder based on data from a sample of two psychiatric hospitals in Beijing. Our study reported that NSSI was experienced by a significant number of patients with depression or bipolar disorder, and NSSI were associated with some factors, including age, employment status, marital status, impulsivity, etc. NSSI not only affects the physical and mental health of the patients, but also places an insupportable burden on the family and society. Some implications for public policy were manifested in this study. The risk of NSSI in patients with depression and bipolar disorder, especially who are young, unemployment, from family with high monthly income, single, impulsive, and have long duration of illness, ACEs should be assessed. Awareness of NSSI should be improved in the family and society. Likewise, training for health care workers and educators are needed, to help them identify and intervene as early as possible [67]. It is necessary to formulate targeted interventions, as well as provide timely social support for patients with NSSI and his family [68]. In terms of methodology, future studies should enrich the access to information, including interviews with patients and their family members, medical records or other objective documents to verify and supplement these reports of patients obtained by using self-rating scales. And future studies can also focus on the relationship between NSSI and its possible risk factors among patients with other types of mental disorders and compare them, rather than focusing on one or two mental disorders alone in order to find the differences.

## Data Availability

All data generated or analysed during this study are included in this published article.

## Abbreviations

NSSI: Non-suicidal self-injury
ACEs: Adverse childhood experiences
ICD-10: The 10th Revision of the International Classification of Diseases
